# Anemia is associated with increased risk of non-vertebral osteoporotic fractures in elderly men: the MrOS Sweden cohort

**DOI:** 10.1007/s11657-022-01130-9

**Published:** 2022-06-23

**Authors:** Hallgerdur Lind Kristjansdottir, Dan Mellström, Peter Johansson, Magnus Karlsson, Liesbeth Vandenput, Mattias Lorentzon, Hans Herlitz, Claes Ohlsson, Ulf H. Lerner, Catharina Lewerin

**Affiliations:** 1 Section of Hematology and Coagulation at the Sahlgrenska University Hospital and Department of Internal Medicine and Clinical Nutrition, Institute of Medicine, Sahlgrenska Academy at the University of Gothenburg, Gothenburg, Sweden; 2 Center for Bone and Arthritis Research (CBAR) at the Department of Internal Medicine and Clinical Nutrition, Institute of Medicine, Sahlgrenska Academy at the University of Gothenburg, Gothenburg, Sweden; 3 Department of Geriatric Medicine, Internal Medicine, Institute of Medicine, Sahlgrenska Academy at the University of Gothenburg, Gothenburg, Sweden; 4 Clinical and Molecular Osteoporosis Research Unit, Department of Clinical Sciences and Orthopedics, Skåne University Hospital (SUS), Lund University, Malmö, Sweden; 5 Mary MacKillop, Institute for Health Research, Australian Catholic University, Melbourne, VIC Australia; 6 Department of Molecular and Clinical Medicine/Nephrology, Institute of Medicine, Sahlgrenska Academy, University of Gothenburg, Gothenburg, Sweden; 7 Department of Drug Treatment, Sahlgrenska University Hospital, Region Västra Götaland, Gothenburg, Sweden

**Keywords:** Anemia, Fractures, iFGF23, Elderly men

## Abstract

***Summary*:**

This study includes 1005 men from the Gothenburg part of the Osteoporotic Fracture in Men Study (MrOS). Included are 66 men with anemia (hemoglobin < 130 g/L). The follow-up time was up to 16 years, and the main results are that anemia is associated with all fractures and non-vertebral osteoporotic fractures.

**Introduction:**

Anemia and osteoporotic fractures are conditions that are associated with increased morbidity and mortality. Clinical studies have suggested that anemia can be used as a predictor of future osteoporotic fractures.

**Method:**

Men from the Osteoporotic Fractures in Men Study (MrOS) Sweden, Gothenburg, with available hemoglobin (Hb) values (*n* = 1005, median age 75.3 years (SD 3.2)), were included in the current analyses. Of these, 66 suffered from anemia, defined as Hb < 130 g/L. Median follow-up time for fracture was 10.1 years and the longest follow-up time was 16.1 years.

**Results:**

Men with anemia had, at baseline, experienced more falls and had a higher prevalence of diabetes, cancer, prostate cancer, hypertension, and stroke. Anemia was not statistically significantly associated with bone mineral density (BMD). Men with anemia had higher serum levels of fibroblast growth factor 23 (iFGF23) (*p* < 0.001) and phosphate (*p* = 0.001) and lower serum levels of testosterone (*p* < 0.001) and estradiol (*p* < 0.001). Moreover, men with anemia had an increased risk of any fracture (hazard ratio (HR) 1.97, 95% CI 1.28–3.02) and non-vertebral osteoporotic fracture (HR 2.15, 95% CI 1.18–3.93), after adjustment for age and total hip BMD, in 10 years. The risk for any fracture was increased in 10 and 16 years independently of falls, comorbidities, inflammation, and sex hormones. The age-adjusted risk of hip fracture was increased in men with anemia (HR 2.32, 95% CI 1.06–5.12), in 10 years, although this was no longer statistically significant after further adjustment for total hip BMD.

**Conclusions:**

Anemia is associated with an increased risk for any fracture and non-vertebral osteoporotic fracture in elderly men with a long follow-up time. The cause is probably multifactorial and our results support that anemia can be used as a predictor for future fracture.

## Introduction

Osteoporosis is an important public health issue because of potential devastating results of osteoporotic fractures. Major osteoporotic fractures are typically defined as fractures of the hip, vertebral body, distal forearm, or humerus [1, 2]. They are associated with increased morbidity and mortality for the individual and are a major economic burden for the society [3]. The risk of osteoporosis is highly dependent on age [4]. Anemia is another public health issue that gets increasingly common in older age [5]. Anemia is defined by the World Health Organization (WHO) as hemoglobin (Hb) < 130 g/L in men and < 120 g/L in women [6]. Blood and bone cells live in close proximity in the bone marrow and all three bone cells, osteoblasts [7], osteoclasts [8, 9], and osteocytes [10, 11], have been implicated to play a role in the hematopoietic stem cell niche and in the differentiation of blood cells. A recent meta-analysis reported that hematopoietic disorders with increased marrow cell proliferation are associated with significant deterioration of bone health, independent of the lineage of the affected blood cells [12]. The association between blood and bone health is also seen in non-proliferative hematopoietic diseases such as Diamond-Blackfan anemia and Fanconi anemia [13]. In recent years, accumulating evidence suggests that anemia is a risk factor for osteoporotic fractures, with most evidence available for non-vertebral fractures in men [14–18]. The primary aim of the present study was to investigate if anemia could predict fracture in a well-defined population-based cohort of ambulatory elderly men, with a long follow-up time.

## Methods

### Study population

The MrOS (Osteoporotic Fractures in Men) study is a prospective, international, multicenter, observational study. The primary objective of the study was to evaluate risk factors for fracture and osteoporosis in elderly men. The overall study design has previously been described [19]. Gothenburg was one of three Swedish sites and recruited 1010 subjects. Men in the age group of 70–80 years living in Gothenburg were randomly identified from national population registries and invited to participate by letter. At the first visit (April 2002–December 2004), subjects answered standardized questionnaires and underwent blood tests and bone mineral density (BMD) measurements. Men with available Hb values were included in the present study (*n* = 1005). The MrOS study was approved by the ethics committee at the University of Gothenburg, Sweden (M 014–01 and LU 611/2012).

### Assessment of covariates

Body weight and height were measured using standard equipment and body mass index (BMI) calculated as weight in kilograms divided by height (in meters) squared (kg/m^2^). Hand grip strength was measured using a Jamar® dynamometer. Areal BMD (aBMD; g/cm^2^) was assessed using DXA with the Hologic QDR 4500/A-Delphi (Hologic, Waltham, MA). aBMD will hereinafter be referred to as BMD. The results for total hip BMD and lumbar spine BMD were standardized with a method that previously has been described [19]. The coefficients of variation (CVs) for the BMD measurements were 0.5–3%. Information regarding general health (hypertension, myocardial infarction, stroke, chronic bronchitis, cancer), falls in the previous year, and smoking was gathered from a standardized questionnaire. Diabetes mellitus was defined as previously diagnosed diabetes, fasting plasma glucose concentration > 7.0 mM/L, or the use of insulin or other hypoglycemic medication. Information regarding prevalent prostate cancer was gathered from the National Prostate Cancer Register of Sweden.

### Blood sampling and analytical methods

Blood samples were collected at the baseline visit, at around 8.00 a.m., following an overnight fast and abstinence from smoking. Hb was analyzed immediately in an automated cell counter (CellDyn 4000; Abbott Diagnostics, Abbott Park, IL, USA), at Sahlgrenska University Hospital, Gothenburg, Sweden. Plasma and serum samples were frozen within 1 h and stored at − 80 °C until required for analysis. Methods regarding plasma/serum concentrations of intact fibroblast growth factor 23 (iFGF23), glucose, osteocalcin, N-terminal propeptide of type 1 collagen (P1NP)*,* testosterone, estradiol, erythropoietin (EPO), C-reactive protein (CRP), intact parathyroid hormone (iPTH), 25(OH)D, and cystatin C, as well as calculation using cystatin C-based formula for estimated glomerular filtration rate (eGFR), have previously been described [20–23]. The serum levels of alkaline phosphatase (ALP), albumin-adjusted calcium, and phosphate were analyzed according to the routine laboratory technique used at Sahlgrenska University Hospital. Hypogonadism was defined as testosterone levels < 8 nmol/L.

### Assessment of fractures

The follow-up time was recorded from the date of baseline visit to the time of fracture, to the end of the study, June 1, 2018, or until death — whichever came first. All Swedish citizens have a unique personal registration number. This enables access to information in X-ray archives concerning the time and the site of fracture. The follow-up time for each fracture type was recorded; namely, any fracture, vertebral fractures, hip fractures, and non-vertebral osteoporotic fractures. Non-vertebral osteoporotic fractures were defined as fractures of the hip, pelvis, proximal humerus, and forearm. Deaths were documented from the National Cause of Death Register that include virtually all deaths in Sweden.

### Statistical analyses

All parametric values are presented as mean with standard deviation (SD), and values that were not normally distributed are presented as median with interquartile range (IQR). Skewed continuous variables were analyzed in the log scale. Differences in means were tested through permutation *t*-test for continuous variables and with the Fisher exact test for dichotomous variables. Since 25(OH)D varies according to season, a *Z*-score was calculated and an expected value of 25(OH)D was attained for each participant according to season [24] and was used in statistical analysis at 25(OH)D values. Hb was analyzed as a dichotomous variable, i.e., Hb < 130 g/L (anemia) or Hb ≥ 130 g/L (not anemia). Using fractures (any, non-vertebral osteoporotic, hip and vertebral) as an event, we calculated the probability of being fracture free during the follow-up period, depending on whether the subject had anemia or not, and plotted them according to the Kaplan–Meier method using a log‐rank test for statistical comparison of the equality in the fractures between the anemic and non-anemic groups. Follow-up time was derived separately for any fracture, vertebral fracture, non-vertebral fracture, and hip fracture outcomes. Patients were followed until fracture, death, or emigration. Poisson regression was used to determine if anemia was associated with risk of fractures after adjusting for other covariates. As Poisson regression uses updated age at each time, it takes partly care of issue of competing risk of mortality. Linear regression analysis was performed to determine if anemia was associated with iFGF23 after adjustment for other covariates. Double-sided tests were used throughout and a *p* value of < 0.05 was regarded as statistically significant. The software used was SAS for Windows, version 9.3 (SAS Institute, Inc. Cary, NC, USA); Stata version 15.1 (StataCorp LLC, College Station, TX, 77,845, USA); and a database and statistics program package developed at the Department of Community Medicine and Public Health, Gothenburg University.

## Results

The current cohort consisted of 1005 men with a mean age of 75.3 years (SD 3.2) and a mean BMI of 26.2 kg/m^2^ (SD 3.5). Hb values were normally distributed. The mean value of Hb was 147 g/L (SD 12). The cohort included 66 men with anemia, of whom 14 had a Hb < 120 g/L. Baseline demographic and laboratory values for the whole study group and the subjects with or without anemia are presented in Table 1. Men with anemia had experienced more falls in the previous year before baseline and had lower hand strength and slower walking speed. Men with anemia had a higher prevalence of hypertension, stroke, diabetes, cancer, and prostate cancer. The 10-year survival rate was 31% in the anemic group and 49% in the non-anemic group (*p* = 0.009). We were neither able to establish a statistically significant difference in total hip BMD between the anemic and the non-anemic group (0.94 vs. 0.96, *p* = 0.159), nor in the difference between bone remodeling markers in anemic and non-anemic subjects. Subjects with anemia had higher EPO, iFGF23, CRP, and phosphate and lower eGFR, while we were not able to show statistical difference regarding PTH, D vitamin, and calcium levels. The association between anemia and iFGF23 was independent of age, EPO, and eGFR (*β* = 6.56, standard error = 2.84, *p* = 0.021).

**Table 1:** Selected demographic data and laboratory values in the total study group and comparison between subjects with anemia (Hb < 130 g/L) and without anemia (Hb ≥ 130 g/L) for the MrOS Sweden cohort, Gothenburg (*n* = 1005); data is presented as mean (SD), median (IQR), or prevalence (%)

	Total study group *n* = 1005	Anemic subjects *n* = 66	Non-anemic subjects *n* = 939	*p* value
Age (years)	75.3 (3.2)	76.0 (3.0)	75.2 (3.2)	0.072
BMI (kg/m^2^)	26.2 (3.5)	25.7 (3.5)	26.2 (3.5)	0.175
Smoking (%)	8	11	8	0.479
Hand grip strength (kg)	43.7 (8.1)	40.1 (8.3)	44.0 (8.1)	< 0.001
Walking speed test (m/s)	1.4 (0.2)	1.3 (0.2)	1.4 (0.2)	0.014
Falls in previous year (%)	16	28	15	0.011
Total body BMD (g/cm^2^)	1.09 (0.11)	1.09 (0.11)	1.09 (0.11)	0.957
Lumbar spine L1–L4 BMD (g/cm^2^)	1.12 (0.20)	1.11 (0.18)	1.12 (0.20)	0.688
Total hip BMD (g/cm^2^)	0.96 (0.14)	0.94 (0.14)	0.96 (0.15)	0.159
Osteocalcin (µg/L)*	25.0 (19.0–31.0)	27.0 (19.0–37.0)	25.0 (20.0–31.0)	0.136
P1NP (µg/L)*	36.8 (29.8–48.8)	39.6 (31.0–56.6)	36.7 (29.7–46.5)	0.073
ALP (µkat/L)*	0.86 (0.71–1.03)	0.92 (0.71–1.22)	0.85 (0.71–1.02)	0.075
25(OH)D (nmol/L)*/**	66 (53–79)	66 (50–80)	66 (53–78)	0.591
Calcium-albumin corrected (nmol/L)	2.2 (0.15)	2.2 (0.17)	2.2 (0.15)	0.369
Phosphate (nmol/L)	1.07 (0.15)	1.13 (0.17)	1.06 (0.16)	0.001
iPTH (pmol/L)*	5.40 (4.20–7.10)	5.50 (4.20–7.50)	5.40 (4.2–7.0)	0.260
iFGF23 (pg/mL)*	42.6 (33.4–54.1)	49.2 (37.6–64.4)	42.0 (32.7–53.4)	< 0.001
EPO (IU/L)*	10.1 (7.8–13.1)	15.8 (11.0–20.1)	9.8 (7.7–12.7)	< 0.001
CRP (mg/L)*	2.03 (1.59–3.02)	2.48 (1.81–7.20)	2.00 (1.58–2.93)	< 0.001
Total estradiol (pmol/L)	76.9 (30.5)	60.2 (31.0)	78.0 (30.1)	< 0.001
Total testosterone (nmol/L)	15.4 (6.5)	11.9 (7.0)	15.6 (6.4)	< 0.001
Hypogonadism (%)	9	24	8	< 0.001
Prostate cancer (%)	9	22	8	< 0.001
Prevalent cancer (%)	16	30	15	0.004
eGFR (mL/min)	71.2 (18.1)	60.9 (20.8)	71.9 (17.8)	< 0.001
Myocardial infarction (%)	15	17	15	0.593
Chronic bronchitis (%)	8	6	9	0.646
Diabetes (%)	15	27	14	0.007
Hypertension (%)	34	47	33	0.040
Stroke (%)	6	14	6	0.014

For continuous variables, *p* values are from permutations *t*-test. For dichotomous variables, *p* values are from the Fisher exact test. *Non-parametric variables log before statistical testing; **for statistical testing, seasonally adjusted D vitamin was used as a variable

### Risk of fractures and anemia

**Fig. 1:**
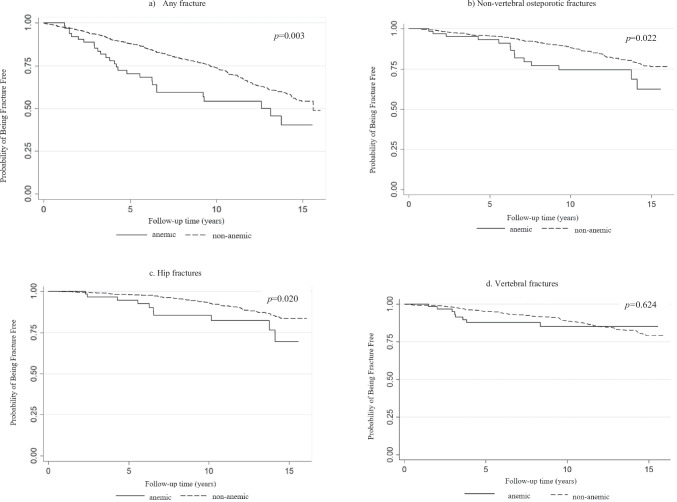
A Kaplan–Meier curve

During a median follow-up time of 10.1 years (SD 4.6, maximal follow-up time 16.1 years), 346 subjects suffered from any fracture: 27 of 66 anemic subjects (41%) and 319 of 938 (34%) of non-anemic subjects. Analysis according to fracture type showed that 162 subjects suffered from a non-vertebral fracture (14 of 66 (21%) anemic and 152 of 938 (16%) non-anemic), 110 from a hip fracture (10 of 66 (15%) anemic and 100 of 938 (11%) non-anemic), and 140 from a vertebral fracture (8 of 66 (12%) anemic and 132 of 938 (14%) non-anemic). Figure 1 depicts a Kaplan–Meier curve and shows the probability of being fracture free in the follow-up period for any fracture, non-vertebral fracture, hip fracture, and vertebral fracture, in the anemic and non-anemic groups. Men with anemia were less likely to be free from any (*p* = 0.003), non-vertebral (*p* = 0.022), and hip fracture (*p* = 0.020) in the follow-up period. For vertebral fractures, the probability of being fracture free was the same for the anemic and non-anemic subjects (*p* = 0.624). We calculated the risk for specific fractures by anemia status with up to 10 and up to 16 years’ follow-up time and adjusted for age and total hip BMD (see Table 2). The HR for any fracture when anemic was 1.97 (95% CI 1.28–3.02) in 10 years and 1.69 (95% CI 1.13–2.52) in 16 years, compared with non-anemic subjects, adjusted for age and total hip BMD*.* Anemia predicted for non-vertebral fractures (HR in 10 years 2.35, 95% CI 1.29–4.27) but not for vertebral fractures (HR in 10 years 1.59, 95% CI 0.77–3.29) after adjustment for age and total hip BMD. Anemic subjects had an increased age-adjusted risk of hip fracture after both 10 and 16 years of follow-up, albeit not significant after further adjustment for total hip BMD.

**Table 2:** Hazard ratio (HR) for fractures in subjects with anemia (Hb < 130 g/L), with 10 years and 16 years of follow-up time for the MrOS Sweden cohort, Gothenburg (*n* = 1005)

	Follow-up time in years	HR for fracture in men with anemia	95% CI
Any fracture			
Adjusted for age	**10**	**2.07**	**1.35–3.19**
	**16**	**1.72**	**1.16–2.57**
Adjusted for age and total hip BMD	**10**	**1.97**	**1.28–3.02**
	**16**	**1.69**	**1.13–2.52**
Non-vertebral fractures			
Adjusted for age	**10**	**2.35**	**1.29–4.27**
	**16**	**1.91**	**1.10–3.30**
Adjusted for age and total hip BMD	**10**	**2.15**	**1.18–3.93**
	**16**	**1.78**	**1.03–3.08**
Hip fractures			
Adjusted for age	**10**	**2.32**	**1.06–5.12**
	**16**	**2.05**	**1.07–3.93**
Adjusted for age and total hip BMD	10	2.08	0.95–4.59
	16	1.82	0.95–3.50
Vertebral fracture			
Adjusted for age	10	1.59	0.77–3.29
	16	1.17	0.57–2.38
Adjusted for age and total hip BMD	10	1.53	0.74–3.16
	16	1.14	0.56–2.33

HR calculated with Poisson regression, *BMD* bone mineral density

Further adjustment was done to assess the risk for all fractures, adjusting for various diseases and conditions (see Table 3). This analysis shows that anemia is independently associated with increased risk for all fractures at 10 and 16 years after adjusting for age, year of birth, smoking, hip BMD, falls, stroke, cancer, diabetes, kidney function, testosterone and estrogen levels, CRP, and EPO.

**Table 3:** Hazard ratio (HR) for any fracture adjusting for multiple covariates in subjects with anemia (Hb < 130 g/L) with 10 years and 16 years of follow-up time for the MrOS Sweden cohort, Gothenburg (*n* = 1005)

	Follow-up time in years	HR for any fracture in men with anemia	95% CI
Base model	10 16	1.98 1.74	1.28–3.05 1.16–2.59
BM + falls	10 16	1.85 1.64	1.18–2.90 1.08–2.48
BM + cancer	10 16	1.84 1.60	1.17–2.90 1.05–2.43
BM + eGFR	10 16	1.98 1.75	1.28–3.07 1.17–2.64
BM + stroke	10 16	1.81 1.59	1.15–2.86 1.04–2.42
BM + diabetes	10 16	1.90 1.65	1.23–2.93 1.10–2.47
BM + testosterone	10 16	1.89 1.66	1.22–2.94 1.11–2.49
BM + estradiol	10 16	1.97 1.72	2.28–3.06 1.14–2.57
BM + CRP	10 16	1.84 1.64	1.19–2.87 1.09–2.46
BM + EPO	10 16	1.62 1.56	1.02–2.60 1.02–2.38

HR calculated with Pearson regression. Base model: age, birth year, smoking, total hip BMD. Continuous data is analyzed per SD and non-parametric data (EPO, CRP) was log before statistical testing. *eGFR* estimated glomerular filtration rate, *CRP* C-reactive protein, *EPO* erythropoietin

## Discussion

In this prospective observational study of 1005 ambulatory elderly men, we found that anemia increased the risk of any fracture independently of age, falls, total hip BMD, and various comorbidities and conditions, even with a long follow-up time of up to 16 years. The increased fracture risk was driven by non-vertebral fractures, where the 10-year risk was increased by 115%, independently of age and total hip BMD. The age-adjusted hip fracture risk was increased by 132%, 10 years from baseline, but, probably due to few fracture events and few anemic men, the risk increase was not statistically significant after adjusted for total hip BMD.

Our results are in agreement with previous studies. A population-based study from Tromsö, Norway, found that anemia was associated with an increased risk of non-vertebral fractures but not for vertebral fractures, in men but not in women, after adjustment for BMD [15]. Similar information was reported from MrOS USA, which only studied men [17]. The National Health Screening Program in Korea reported an association between anemia and both vertebral and non-vertebral fractures, although the risk increase was higher for non-vertebral fractures [16]. The Korean cohort included 72,131 subjects, while our cohort, as well as the MrOS USA and the Tromsö cohorts, was smaller, thus making it harder to show a statistically significant risk difference. Furthermore, no adjustments were made for BMD in the Korean cohort [15–17]. Conflicting results are published on the association between Hb values or anemia and fracture in women [14–16].

In agreement with other studies, we showed that anemia is associated with increased frailty, morbidity, and mortality [5, 25]; however, the risk for fractures was independent of baseline falls and various diseases and conditions. Men with anemia had a higher prevalence of diabetes, as previously described [26]. Diabetes is known to be associated with increased risk of osteoporotic fractures [27], as well as being associated with hypogonadism [28]. The prevalence of prostate cancer was markedly increased in the anemic population compared to the non-anemic population, as well as the prevalence of hypogonadism. Hypogonadism is associated with prostate cancer, partly due to its treatment with androgen deprivation therapy (ADT), as well as the prostate cancer in itself being associated with lower testosterone values [29]. Both low testosterone and low estradiol in men are known to be associated with decreased BMD and increased risk for fractures [19, 30], as well as being associated with anemia, in our, as well as previous studies [23, 31]. Chronic kidney disease (CKD) and inflammation are known causes of secondary anemia and have both been associated with increased risk of fracture [32, 33], which is in agreement with our results. The mechanism by which anemia increases fracture risk is probably multifactorial and it is likely that anemia, to some extent, serves as a proxy for the underlying disease burden. Another mechanisms through which anemia might be associated with fracture risk is via higher phosphate and iFGF23, both related to anemia in our study. Both are associated with decreased renal function [34]. Hyperphosphatemia has been related to anemia [35] and increased fracture risk [36]. FGF23 is produced by osteocytes/blasts and is a known effector of bone mineralization, but our group has previously shown that Hb as a continuous variable correlates with iFGF23 [37]. We further demonstrated that anemia was associated with iFGF23, independently of renal function, EPO, and age. Contradicting results are published on the association between FGF23 and fracture risk [38, 39]. Another possible mechanism through which anemia affects bone is via elevated levels of EPO in the blood. Our group has previously shown that increased levels of plasma EPO are associated with increased risk of fracture in elderly men, although most noticeable for vertebral fractures [21]. EPO production is stimulated via hypoxia and bone homeostasis is effected by oxygen tension. Preclinical studies have shown that hypoxia has a negative effect on bone both by inhibiting osteoblast formation and stimulating osteoclast formation [40]. Clinical studies have shown that short-term exposure of high altitude, which is associated with hypoxia, can lead to decreased BMD [41, 42], and hypoxia during sleep in elderly men leads to increased risk for falls and fractures [43]. Thus, it is possible that anemia increased fracture risk partly because of relative hypoxia. In the bone marrow, EPO controls the proliferation and differentiation of erythrocytes [44]. Several preclinical studies have shown that EPO has a detrimental effect on bone mass in adult rodents, but other studies in growing mice and using traumatic models have reported a stimulation of bone formation by EPO [45]. Our group has recently shown that EPO is associated with increased BMD in elderly men [21]. It has been suggested that EPO is a cross-link between the hematopoietic stem cell niche and bone cells. Osteoblasts can produce EPO, which results in the expansion of the hematopoietic stem cell niche and is associated with selective expansion of erythroid cells [46].

In our present study on anemia, we were not able to establish a statistically significant association with lower BMD. The reason for this can be either a real lack of association or a lack of power in our study owing to the small number of men with anemia. The latter would be supported by the loss of statistical significance after adding BMD in multivariate Poisson regression for hip fractures. Our group has recently shown that Hb analyzed as a continuous variable is associated with total hip BMD after adjustment for age and BMI. When adjusted for estradiol or osteocalcin, the association was no longer significant [47]. Hb has been associated with lower bone mass in two Italian cohort studies [48, 49]. In these studies, bone mass was measured with peripheral quantitative computed tomography (pQCT) or ultrasound-derived *T*- and *Z*-score, respectively. In another study where BMD has been measured with DXA, no association between anemia and cross-sectional BMD was seen. In that study, an association between fast annual change in BMD and anemia was seen [50]. It is therefore possible that anemia is associated with bone mass in a way that was not captured by a single DXA or anemia is associated with bone fragility not captured by DXA. In a recent meta-analysis by Steer et al., an association between bone marrow cellularity evaluated with a trephine biopsy, and bone density change was seen, whereas no association was seen with Hb measurement and bone density change [12]. Peripheral blood cell count may not be the best way to evaluate hematopoiesis, rather a trephine biopsy would be preferable; however, this was not collected in the MrOS cohort.

An important strength of our study is the long follow-up time for fractures and a relatively large cohort. The fracture data are reliable owing to the unique personal registration number of all Swedish citizens, making it possible to access information in X-ray archives about the time and the site of fracture. Fasting and standardized sampling of data were used for all blood samples. We acknowledge several limitations in our study. First, it is a cross-sectional study, with only single measurements of blood variables and BMD. A significant limitation is that the anemic group only contains 66 subjects. Thus, the total amount of events was few in the anemic group which makes statistical analysis more vulnerable and true associations can be missed. Since this is an observational study, causality cannot be determined. Although multiple covariates were tested, we cannot rule out the possibility of residual confounding.

In conclusion, our findings indicate that anemia, after controlling for multiple factors, is predictive for any fracture and non-vertebral osteoporotic fractures in elderly men. The underlying mechanism is probably multifactorial but measuring Hb might be useful for long-term risk prediction for fractures.
